# Inducible general knockout of Runx3 profoundly reduces pulmonary cytotoxic CD8^+^ T cells with minimal effect on outcomes in mice following influenza infection

**DOI:** 10.3389/fimmu.2022.1011922

**Published:** 2022-10-07

**Authors:** Qin Hao, Suman Kundu, Sreerama Shetty, Torry A. Tucker, Steven Idell, Hua Tang

**Affiliations:** Department of Cellular and Molecular Biology, The University of Texas Health Science Center at Tyler, Tyler, TX, United States

**Keywords:** *Runx3*, influenza, lung, CD8^+^ T cells, macrophages, dendritic cells

## Abstract

Respiratory viruses pose a continuing and substantive threat to human health globally. Host innate and adaptive immune responses are the critical antiviral defense mechanisms to control viral replication and spread. The present study is designed to determine the role of transcription factor Runx3 in the host immune response to influenza A virus (IAV) infection. As Runx3 is required for embryonic development, we generated an inducible *Runx3* global knockout (KO) mouse model and found that *Runx3* KO in adult C57BL/6 mice minimally affected thymic function under normal conditions and survival was at least 250 days post *Runx3* deletion. We applied the mouse model to IAV infection and found that *Runx3* KO resulted in a huge reduction (>85%) in numbers of total and antigen-specific pulmonary CD8^+^ cytotoxic T cells during IAV infection, while it had a minor effect on pulmonary generation of CD4^+^ T cells. To our surprise, this general KO of *Runx3* did not significantly alter viral clearance and animal survival following IAV infection. Interestingly, we found that *Runx3* KO significantly increased the numbers of pulmonary innate immune cells such as macrophages and neutrophils and the production of pro-inflammatory cytokines during IAV infection. We further found that Runx3 was strongly detected in CCR2^+^ immune cells in IAV-infected mouse lungs and was induced in activated macrophages and dendritic cells (DCs). As pulmonary CD8^+^ cytotoxic T cells play a central role in the clearance of IAV, our findings suggest that *Runx3* KO may enhance host innate immunity to compensate for the loss of pulmonary CD8^+^ cytotoxic T cells during IAV infection.

## Introduction

Respiratory viruses affect all age groups and cause considerable morbidity and mortality with a formidable toll on world health and economics (1–4). Respiratory viruses pose a continuing and substantive threat to human health globally. Respiratory viral infection triggers host innate and adaptive immune responses, which are the critical antiviral defense mechanisms to control virus replication and spread (5–8). This study is to identify the critical host immune modulators following influenza A virus (IAV) infection.

The RUNX transcription factors play pivotal roles in normal embryonic development, neoplasia and immunity (9, 10). In mammals, the RUNX family consists of three members including Runx1, Runx2 and Runx3, and each RUNX member has a distinct set of functions (11). Runx1 and Runx2 are essential for hematopoiesis and osteogenesis, respectively (12, 13). Runx3 is required for embryonic development and involved in thymopoiesis together with Runx1 (13). The overall gene size and organization between mouse and human *Runx3* are similar (14). C57BL/6 and BALB/c mice with a general knockout (KO) of *Runx3* died soon after birth (15, 16); whereas some ICR and MF1 (17) background outbred mice with a general *Runx3* KO survived, but had developmental defects such as a decreased body weight (50% of control mice) (18, 19). Currently, the general role of Runx3 in adulthood is not clear. On the other hand, T or NK cell-specific *Runx3* KO mice are viable (20–22). By using the T cell-specific *Runx3* KO mouse model, it has been demonstrated that Runx3 is required for the differentiation, recruitment and local expansion of CD8^+^ T effector/memory cells and for the clearance of lymphocytic choriomeningitis virus (LCMV) (21, 23). Similarly, Runx3 is also required for the differentiation and local expansion of NK cells in response to mouse cytomegalovirus infection, which was demonstrated in NK cell-specific *Runx3* KO mice (22). In outbred (ICR and MF1) mice with a general *Runx3* KO, studies have shown that Runx3 is required for TGFβ signaling and function in dendritic cells (DCs). KO of *Runx3* promoted DC maturation and its potency to stimulate CD4^+^ T cell proliferation (18). KO of *Runx3* also promoted pulmonary DC migration to thoracic lymph nodes by lipopolysaccharide (LPS) treatment (19). Moreover, KO of *Runx3* in plasmacytoid DCs (pDCs) promoted DC maturation by CpG treatment and enhanced skin fibrosis by bleomycin in mice (24). In humans, Runx3 expression is often reduced in tumor tissue cells including immune cells and in pDCs of systemic sclerosis patients (9, 24). Conversely, Runx3 expression is increased in CD4^+^ T cells from patients with psoriasis (25).

We previously showed that Runx3 was induced by IAV infection and double-stranded RNA (dsRNA) and meditated cell apoptosis in human airway epithelial cells (26). We also found that Runx3 mediated interferon-γ (IFNγ)-promoted dsRNA-induced apoptosis in human airway epithelial cells (27). A recent report showed that Runx1 could also be induced by IAV infection and inhibited host antiviral response through attenuating type I IFN signaling in A549 human alveolar adenocarcinoma epithelial cells (28). These data suggest that Runx3 may impact host immune responses to IAV infection. However, the general role of Runx3 in the pathogenesis of respiratory viral infection *in vivo* is currently not clear. To address this gap in knowledge, we generated an inducible *Runx3* general KO mouse model and applied the mouse model to study the role of Runx3 in host immune responses to IAV infection.

## Materials and methods

### Antibodies and reagents

Antibodies specifically against Runx3 (no. 9647 and 13089) were from Cell Signaling Technology (Beverly, MA, USA). Vinculin (no. V9131) and actin (no. A4700) antibodies, tamoxifen (no. T5648) and LPS (*Escherichia coli* 0111:B4) were from Sigma (St. Louis, MO, USA). Antibody against IAV nucleoprotein (NP, no. GTX125989) was from GeneTex (Irvine, CA, USA). Recombinant mouse interlulin-4 (IL-4, no. 574304), macrophage-colony stimulating factor (M-CSF, no. 576404), and granulocyte/macrophage-colony stimulating factor (GM-CSF, no. 576304), FITC anti-mouse CD192 (CCR2, no. 150607) and TruStain fcX (anti-mouse CD16/32, no. 101319) antibodies were from BioLegend (San Diego, CA, USA). Alexa Fluor-568 goat anti-mouse secondary antibody (no. A-11004) and recombinant mouse interferon-γ (IFNγ, no. 34-8311) were from Thermo Fisher Scientific (San Diego, CA, USA). Polyinosinic-polycytidylic acid (poly(I:C)) (no. tlrl-pic) was from *In vivo*Gen (San Diego, CA, USA). Recombinant mouse tumor necrosis factor-α (TNFα, no. 410-MT) was from R & D Systems (Minneapolis, MN, USA). Vector MOM immunodetection kit (no. PK-2200), Vector NovaRED substrate (no. SK-4800), Vector hematoxylin (no. H-3401), VectaMount permanent mounting medium (no. H-5000), and Vectashield hardset antifade mounting medium with DAPI (no. H-1500) were from Vector Laboratories (Burlingame, CA, USA). Clarity Western ECL substrate was from Bio-Rad (Hercules, CA, USA).

### Generation of inducible *Runx3* general KO mouse model

All animal experiments were approved by the Institutional Animal Care and Use Committee at the University of Texas Health Science Center at Tyler. Runx3 is essential for normal embryonic development and *Runx3* KO mice on the background of C57BL/6 and BALB/c died soon after birth (15, 16). To assess the general role of Runx3 in adult mice, we generated an inducible *Runx3* global KO mouse model using Cre/Lox technology. We first recovered the conditional ready *Runx3^f/f^* mice with exon 4 flanked by loxP sites from cryopreserved condition (B6.129P2-*Runx3^tm1Itan^*/J, #008773, Jackson Laboratory, Bar Harbor, ME). We crossed *Runx3^f/f^* mice with *CreER^T2^-ROSA26* mice (B6.129-*Gt(ROSA)26Sor^tm1(cre/ERT2)Tyj^*/J, #008463, Jackson Laboratory) for four generations and successfully obtained the desirable genotype of inducible *Runx3* KO mice. Animal genotyping was performed as described previously (20, 29). To induce *Runx3* KO in all the cells and tissues, the inducible *Runx3* KO (*Runx3^f/f^:ROSA26-ERCre^+^*) and littermate control (*Runx3^f/f^:ROSA26-ERCre^-^*) mice at 6 to 8 weeks old were administered with tamoxifen (200 mg/kg body weight) dissolved in corn oil *via* intraperitoneal (i.p.) injection using 23G needles every two days for eight days as described previously (30). Two weeks after tamoxifen treatment, the efficiency of *Runx3* KO was verified by PCR and Western blot analyses. At least one month post tamoxifen treatment, sex- and age-matched littermate control and Runx3 KO mice were used for studies.

### DNA isolation and PCR

Total DNA from mouse tail was isolated by using the DNeasy Blood and Tissue kit (no. 69504) according to the manufacturer’s protocol (Qiagen, Valencia, CA, USA). Runx3 primers were 5′-TAT CCC TCT CTG GGC CTT CT-3′ (forward) and 5’-GGA AAC TGA GTC CAG CCA AG-3’(reverse). PCR conditions were 94°C for 5 min, followed by 25 cycles of 94°C for 30 s, 62°C for 1 min, and 72°C for 1 min, followed by 72°C for 10 min.

### Western blot analysis

Western blot analysis was performed as we described (31). Briefly, cells were washed and then lysed on ice in Nonidet P-40 lysis buffer (25 mM Tris-HCl, pH 7.5, 1% Nonidet P-40, 150 mM NaCl, 10 mM NaF, 1 mM Na3VO4, 1 mM phenylmethylsulfonyl fluoride, 10 mg/ml each leupeptin and aprotinin). Total protein concentrations were determined by Bradford method using Bio-Rad protein assay kit (no. 5000001, Hercules, CA, USA). Equal amounts of whole cell lysates were subjected to SDS-PAGE and transferred to polyvinylidene difluoride membranes. The membranes were blocked and then incubated 1 h at room temperature with a validated primary Runx3 antibody (1:1000, no. 9647, Cell Signaling Technology, Danvers, MA), followed by 1 h incubation with an anti-rabbit HRP-conjugated secondary antibody (no.7074, Cell Signaling Technology, Beverly, MA). Labelled proteins were detected with Bio-Rad Clarity Western ECL substrate (no. 1705061, Hercules, CA). To verify equal sample loading, the same membranes were incubated with a mild antibody stripping solution for 20 min at room temperature (no. 2502, Sigma, St. Louis, MO) and reprobed with actin (1:1000, no. A4700) or vinculin (1:10,000, no. V9131, Sigma, St. Louis, MO) antibodies, followed by 1 h incubation with an anti-mouse HRP-conjugated secondary antibodies (no. 7076, Cell Signaling Technology, Beverly, MA).

### IAV infection and animal experiments

Wild type C57BL/6J mice were obtained from Jackson Laboratory (Bar Harbor, ME). Wild type C57BL/6J mice at 6–8 weeks old, sex- and age-matched *Runx3* KO and littermate control mice on C57BL/6 genetic background were anesthetized by i.p. injection of ketamine (100 mg/kg) and xylazine (8.5 mg/kg). These mice were then administered *via* intranasal instillation of 35 μl sterile saline or 35 μl saline containing 1 to 1.5 LD_50_ (~ 330-500 plaque-forming units (pfu)) H1N1 PR/8/34 (Charles River, Wilmington, MA). Viral dilution from stock with saline and animal infection were performed in a class II biosafety cabinet, and the infected mice were housed in a designated BSL2 room. Personnel conducting the experiments were rigorously trained and protected by use of PPE (gloves, masks, gowns and shoe covers). Working surfaces were decontaminated after experiments with 70% ethanol, and hand-washing with an alcohol gel was done after experiments according to the BSL2 research safety and work practices manual. Mice were observed daily for signs of distress by monitoring general appearance, respiratory difficulties, body weight loss and animal survival. Mice which lost more than 30% of their initial body weight were humanely euthanized by CO_2_ inhalation followed by cervical dislocation. On days 3, 6, 9, and 12 post-infection (pi), the infected *Runx3* KO and control mice were anesthetized with injectable anesthesia and then sacrificed by exsanguination to harvest tissues. For the collection of bronchoalveloar lavage fluids (BALFs), a small incision of trachea was made to insert a 21G cannula which was then firmly fixed. The lungs were lavaged with 0.75 ml saline six times to collect BALFs. The first two BALFs were combined for cytokine ELISA assays, and lung immune cells from six BALFs were combined for flow cytometry analysis for each mouse. After that, the lungs were perfused and harvested for experiments. In some cases, the lungs were directly perfused without collecting BALFs. For IAV infected wild type mice, animals were euthanized and lungs were perfused and harvested on designated time points. Whole lungs were fixed in 10% formalin for at least 48 h and formalin-preserved lungs were processed and embedded in paraffin *via* standard procedures.

### Virus titration

The infectious IAV viruses in BALFs were quantified by using 50% tissue culture infective dose (TCID_50_) assay in Madin-Darby Canine Kidney (MDCK) cells as described previously (32). This endpoint dilution assay quantifies the amount of virus required to kill 50% of infected cells or to produce a cytophathic effect in 50% of inoculated cells. MDCK cells (ATCC, Manassas, VA) were cultured in 96-well plates with complete EMEM growth medium until subconfluent, then infected with samples containing IAV with a serial 10-fold dilutions. After 72 h, cytopathic effects that include rounding of the infected cells, cell fusion and detachment from culture plate were observed and quantified under a microscope, and the TCID_50_ was calculated by the Reed-Muench method (33).

### Immunohistochemistry

Immunohistochemistry was performed as we described previously (34). Briefly, mouse lung paraffin sections (5 μm) were deparaffinized, rehydrated and then subjected to antigen retrieval by incubating the sections at 95°C for 10 min in 10 mM sodium citrate buffer (pH 6.0). The slides were then treated with 3% H_2_O_2_ for 5 min and washed in phosphate-buffered saline (PBS) containing 0.1% Tween-20. A M.O.M (mouse on mouse) immunodetection kit was used for immunohistochemical staining according to the manufacturer’s instructions (Vector Laboratories, Burlingame, CA). Lung sections were incubated overnight at 4°C with a validated primary Runx3 monoclonal antibody (1:100) or NP antibody (1:1000). Non-immune normal isotype control antibody served as the negative experimental control. After incubation with biotinylated secondary antibody and ABC reagents, all lung sections were incubated with peroxidase substrate Vector NovaRed for an equal amount of time to allow for suitable staining. The slides were counterstained with hematoxylin followed by a brief bluing with 0.45% NH_4_OH in 70% ethanol, mounted with VectaMount medium, examined and photographed using an Olympus BX41 microscope imaging system.

### Flow cytometry

BALF cells, thymic and lung single cell-suspensions were used for flow cytometry analyses. Cells were resuspended in cell staining buffer (no. 420201, BioLegend, San Diego, CA) and blocked with TruStain FcX (anti-mouse CD16/32) antibody specific for FcγR III/II (no. 101320, BioLegend) for 10-15 min on ice. Cell surface staining was performed by incubating the cells in the dark for 30 min at 4°C with following specific fluorochrome-conjugated antibodies or isotype controls (BioLegend unless otherwise specified): Alexa Fluor-488 anti-CD45, PE anti-CD3, APC anti-CD4, FITC anti-CD4, PE-Cy7 anti-CD8α, PE anti-F4/80, APC anti-CD11b, Alexa Fluor-488 anti-CD11b, PE/Cy7 anti-CD11c, PE anti-Ly6G, or FITC isotype control antibodies as well as PE-conjugated tetramer specific for H-2Db IAV NP_366-374_ (MBL International Corporation, Woburn, MA). After washing, stained cells were acquired in an Attune NxT flow cytometer that can detect up to 14 colors (Thermo Fisher Scientific) and data were analyzed using FlowJo software v10.6.2.

### Cytokine ELISA

IL-6, IL-8 and IFNβ were determined using ELISA kits from R &D Systems (Minneapolis, MN, USA) according to the manufacturer’s instructions.

### Immunofluorescence microscopy

Immunofluorescence staining was performed as we described previously (34). Briefly, mouse lung paraffin sections (5 μm) were deparaffinized, rehydrated and then subjected to antigen retrieval by incubating the sections at 95°C for 10 min in 10 mM sodium citrate buffer (pH 6.0). The lung tissue sections were then blocked and incubated overnight at 4°C with Runx3 (1:100) mouse monoclonal antibody and/or FITC conjugated CD192/CCR2 rat monoclonal antibody (10 μg/ml). After washing by PBS with 0.1% Tween-20, the lung sections were incubated 25 min with Alexa Fluor-568 goat anti-mouse secondary antibody (1:200) at room temperature in the dark. The sections were then washed and mounted with Vectashield anti-fade mounting medium with DAPI. Fluorescence was visualized and captured using a Nikon fluorescence imaging system. Adobe Photoshop CS6 software was used for image processing.

### Isolation of lung innate immune cells

To prepare lung single-cell suspension, the perfused whole lungs from IAV-infected mice on day 5 pi were minced and incubated with RPMI-1640 medium containing 0.5% fetal bovine serum (FBS), 1 mg/ml type I collagenase (Worthington, Lakewood, NJ) and 30 Units/ml DNase (Promega, Madison, WI) for 45 min at 37 °C. This was followed by passing through 100- and 70-µm nylon filters. After lysis of red blood cells, lung cells were resuspended in MACS buffer containing PBS, 0.5% bovine serum albumin (BSA) and 2mM EDTA. CD11b^+^, CD11c^+^ and F4/80^+^ innate immune cells were subsequently isolated by using antibody-conjugated magnetic microbeads and MACS LS columns according to the manufacturer’s instructions (Miltenyi Biotec, Auburn, CA). All procedures were performed in a class II biosafety cabinet, and personnel conducting the experiments were trained and protected properly. Wastes containing viruses were removed to a container filled with 10% bleach for decontamination. The working surface after experiments and any contaminated equipment were decontaminated with 70% ethanol according to the BSL2 institutional research safety and work practices manual.

### Primary cell culture

Bone marrow derived macrophages (BMDMs) and dendritic cells (BMDCs) were prepared from the femur and tibia of C57BL/6J mice (Jackson Laboratory, Bar Harbor, ME) as we described previously (34, 35). Briefly, mice were euthanized, after which tibias and femurs were removed under sterile conditions, and the bone marrow was flushed out of the cavity by using a 5-ml syringe. After lysis of red blood cells, the resultant cells were cultured in RPMI-1640 medium supplemented with 10% FBS, 10 ng/ml M-CSF, and 50 μg/ml penicillin/streptomycin to generate BMDMs. Complete culture medium was changed on day 3 and day 5, and BMDMs were fully differentiated on day 7. For the generation of BMDCs, GM-CSF and IL-4 were added into RPMI-1640 medium with 10% FBS to final concentrations of 20 ng/ml and 10ng/ml respectively, and the complete culture medium was changed on days 3 and 5. On day 7, the semi-suspended and loosely-adherent cells were collected and pooled and used as BMDCs. For cell treatment, freshly isolated BMDMs and BMDCs were cultured in 12-well plates and treated with IFNγ (50 ng/ml), TNFα (10 ng/ml), IL-4 (10 ng/ml), LPS (100 ng/ml) or poly(I:C) (0.5 μg/ml) for 24 h in RPMI-1640 medium supplemented with 2.5% FBS.

### Statistical analysis

Data are expressed as mean ± SE. Statistical analyses were performed using GraphPad Prism 9 (GraphPad Software, La Jolla, CA, USA). Unpaired Student’s t test was used to analyze data between means of two groups. Two-way ANOVA followed by Sidak multiple comparisons were performed to compare a response with 2 factors. Differences with p<0.05 were considered statistically significant.

## Results

### Generation and verification of an inducible *Runx3* general KO mouse model

**Figure 1:**
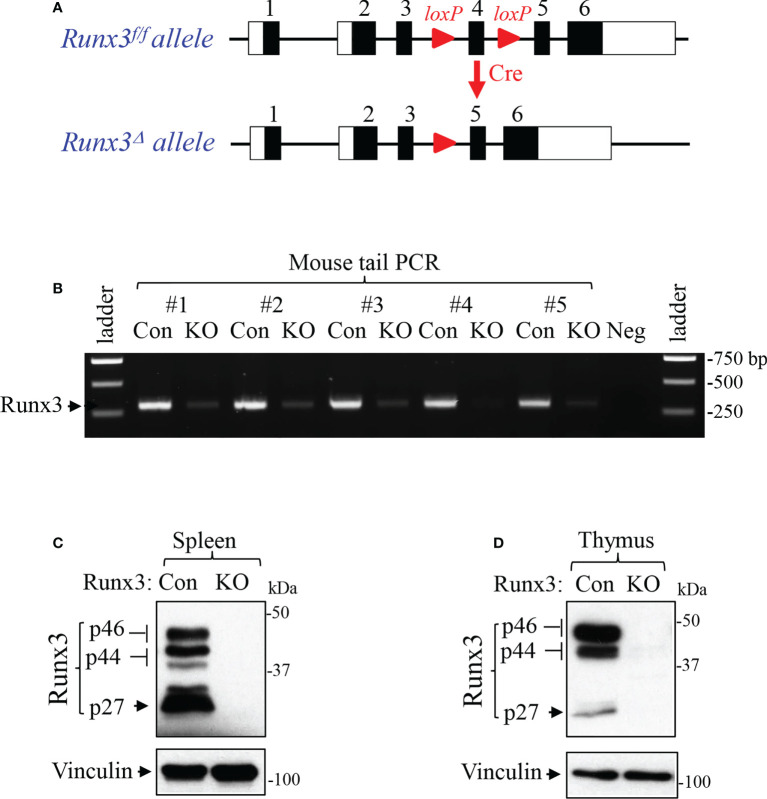
Generation and verification of an inducible *Runx3* general KO mouse model. **(A)** Schematic illustration of the *Runx3* ^f/f^ and conditional *Runx3* KO alleles. **(B)** Verification of *Runx3* deletion by mouse tail PCR in 5 paired sex- and age-matched littermate control (Con) and *Runx3* KO mice two weeks after tamoxifen treatment. Neg: negative control. **(C, D)** Verification of *Runx3* KO by Western blot analysis in spleen **(C)** and thymus **(D)** from uninfected control (Con) and *Runx3* KO mice two weeks after tamoxifen treatment. Equal amounts of cell lysates were subjected to Western blotting with Runx3 antibody. Results are representative of the findings of three independent experiments.

To determine the role of Runx3 in adulthood, we generated an inducible *Runx3* global KO mouse model using Cre/Lox technology. When crossed with *CreER^T2^-ROSA26* mice, the floxed exon 4 of *Runx3* is expected to be deleted to induce a frameshift mutation and trigger nonsense-mediated decay of *Runx3* mutant, causing *Runx3* disruption (**Figure 1A**). As shown in **Figure 1B**, PCR analysis from mouse tail DNA revealed that exon 4 of *Runx3* gene was near completely deleted compared to littermate control mice two weeks after tamoxifen treatment. However, Western blot analysis indicated that Runx3 protein bands were not detected in spleen and thymus from *Runx3* KO mice by an antibody against the N-terminal region of Runx3 (**Figures 1C, D**). These data suggest that the efficiencies of Runx3 gene excision by Cre enzyme could be different among different tissues and cell types. We thus used mice at least one month post tamoxifen treatment for experiments.

### A general *Runx3* KO in adult mice has a minor effect on animal survival and thymic function

**Figure 2:**
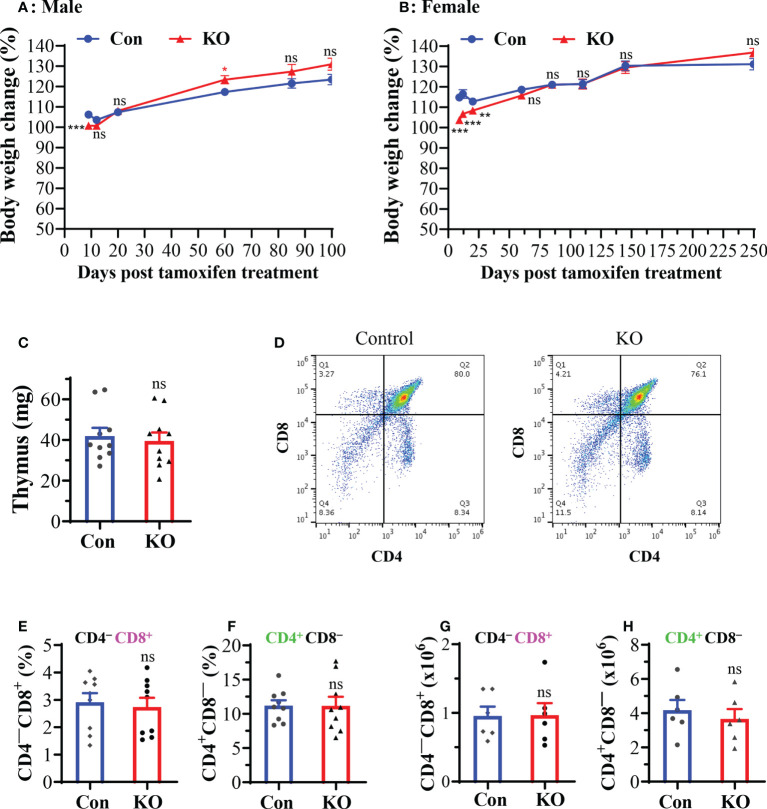
A general *Runx3* KO in adult mice has a minor effect on animal survival and thymic T cell generation under normal conditions. **(A, B)** Body weight changes in age-matched male **(A)** and female **(B)** littermate control (Con) and *Runx3* KO mice after tamoxifen treatment. Mouse body weights were measured from days **(D)** 9 to d250 post last tamoxifen treatment, and relative changes were calculated by comparison to the initial body weight before tamoxifen treatment and are presented as means ± SE (n = 6 to 22 each group). In panel A: d9 to d20, n=19-22; d60 to d100, n=8-13. In panel B: d9 to d145, n = 12-16; d250, n = 6. **(C–H)** *Runx3* KO in adult mice has a minor effect on thymic function. **(C)** Thymus weights of control and *Runx3* KO mice (n = 10 each group) **(D)** Representative thymic FACS plots evaluated one month after tamoxifen treatment are shown for control and *Runx3* KO mice. **(E–H)** The cell frequencies and absolute numbers of single CD8^+^ **(E, G)** or CD4^+^ **(F, H)** positive T cells in thymus are shown in the bar graphs. Results are presented as means ± SE (n = 6-9). Unpaired Student’s t test was performed in **(A–H)**. *p<0.05; **p<0.01; ***p<0.001 vs control mice. NS, not statistically significant.

We found that male mice with inducible *Runx3* KO gained less weight on day 9 post tamoxifen treatment compared to control mice, but essentially showed no significant difference after days 12 to 100 (**Figure 2A**). Most male mice had been used for IAV infection experiments before day 100 post tamoxifen treatment. Female mice with *Runx3* KO relatively gained less weight from days 9 to 20 post tamoxifen treatment, but showed no significant difference after days 60 to 250 (**Figure 2B**). Moreover, we found that inducible *Runx3* KO did not significantly affect thymus weight (**Figure 2C**), the frequency and absolute cell numbers of single CD4^+^ or CD8^+^ positive T cells in the thymus **(** **Figures 2D–H**). These results indicate that *Runx3* KO in adult mice has a minor effect on animal survival and thymic T cell generation under normal conditions.

### General KO of *Runx3* in adult mice slightly reduces mortality in response to IAV infection

**Figure 3:**
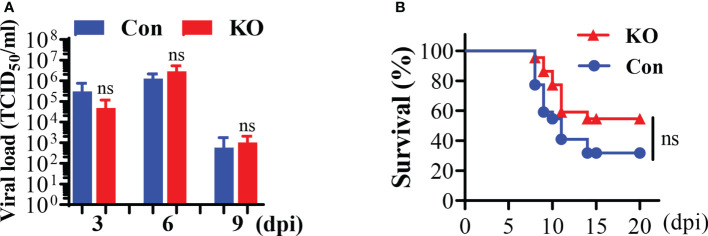
A general *Runx3* KO in adult mice has a minor effect on viral clearance and mortality by IAV infection. **(A)** KO of *Runx3* has a minor effect on IAV viral clearance. Sex- and age-matched littermate control (Con) and *Runx3* KO (KO) mice were infected with 1 LD50 of H1N1 PR8 strain. BALFs were subjected to infectious virus titer assay in MDCK cell and presented as TCID_50_/ml (n=5-6). **(B)** KO of *Runx3* has no significant effect on the mortality by IAV infection. Sex-and age-matched control (n=22) and *Runx3* KO (n=22) mice were infected with 1.35 LD50 of H1N1 PR8 strain and animal survival was assessed over a period of 20 days pi. Animals were monitored until death. Two control mice and one KO mouse had weight loss of more than 30%, at which point they were euthanized and counted as dead. Student’s t test was performed in **(A)** and Log-rank (Mantel-Cox) test was performed in **(B)**. NS, no significance.

We applied the *Runx3* general KO mouse model to study its role in IAV infection. We determined the effect of *Runx3* KO on IAV clearance in lungs. We found that a general KO of *Runx3* had a minor effect on IAV viral clearance through analysis of lung infectious viral titers (**Figure 3A** **)**. We further found that littermate control mice started dying from day 8 to day 11 post IAV infection, while *Runx3* KO mice died mainly from day 9 to day 11 pi (**Figure 3B**). At day 14 pi, two mice from littermate control group and one from *Runx3* KO group died. The overall mortality rate for the control mouse group was 68%, while 46% for *Runx3* KO mice. These data indicate that *Runx3* KO mice tended to have reduced mortality caused by IAV infection compared with littermate control mice, although the effect was not statistically significant.

### Runx3 is mainly detected in immune cells in IAV-infected mouse lungs

**Figure 4:**
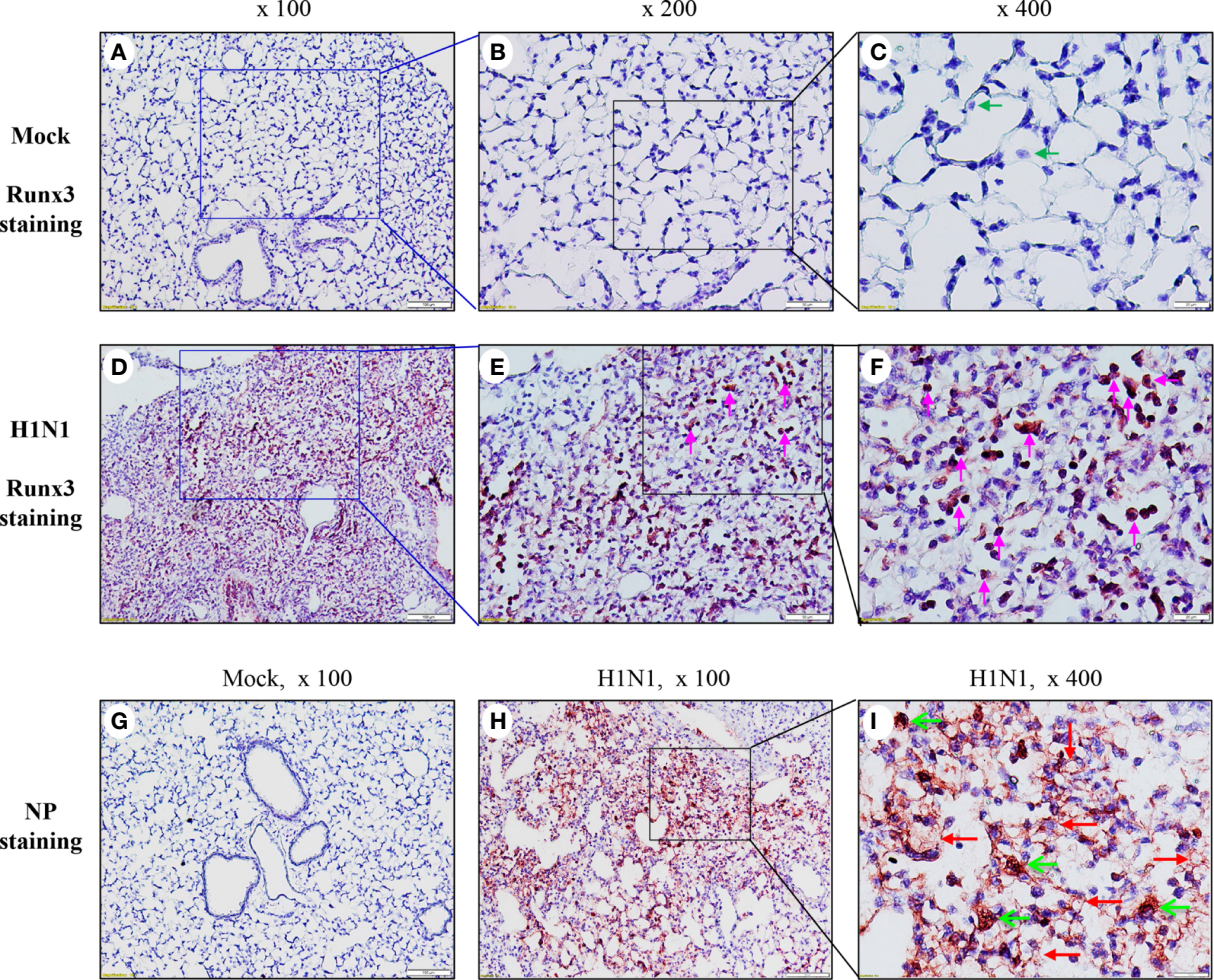
Runx3 is mainly detected in immune cells in IAV-infected mouse lungs.**(A–F)** Immunohistochemistry analysis of Runx3 in mock control and IAV-infected mouse lungs. Lung sections from saline-treated (Mock, **A–C**) or H1N1-infected mice on day 7 pi **(D–F)** were subjected to immunostaining with a Runx3 monoclonal antibody by using Vector M.O.M immunodetection kit (n=6 each group). Positive immunoreactivities (red) were mainly detected in immune cells of IAV-infected mouse lungs (**D–F,** pink arrows). The regions indicated in **(A, D)** (magnification, x100) are shown at higher magnification in **(B, E)** (x200) and in **(C, F)** (x400). **(G–I)** Immunohistochemistry analysis of IAV NP in mock control and IAV-infected mouse lungs. Lung sections from saline-treated (Mock, **G**) or H1N1-infected mice on day 7 pi **(H, I)** were subjected to immunostaining with a specific NP polyclonal antibody (n=3/group). Positive immunoreactivities (red) were detected in epithelial (red arrows) and immune cells (green arrows) of IAV-infected mouse lungs **(H, I)**.

To understand the role of Runx3 in host response to IAV infection, we performed immunohistochemistry analysis to map the general expression of Runx3 in infected mouse lungs. As shown in **Figures 4A–C**, Runx3 immunoreactivity was barely detected in control wild type C57BL/6J mouse airway and alveolar epithelial cells as well as alveolar macrophage-like cells (green arrows). In contrast, strong Runx3 immunoreactivity (red) was readily observed in the infiltrating mononuclear immune cells in IAV-infected mouse lungs on day 7 pi (**Figures 4D–F**, pink arrows). IAV NP immunoreactivity was also readily detected in the infected mouse lungs, confirming the presence of IAV infection (**Figures 4G–I**). These findings indicate that Runx3 is mainly detected in immune cells in IAV-infected mouse lungs.

### KO of *Runx3* in adult mice profoundly reduces CD8^+^ cytotoxic T cells in IAV-infected lungs

**Figure 5:**
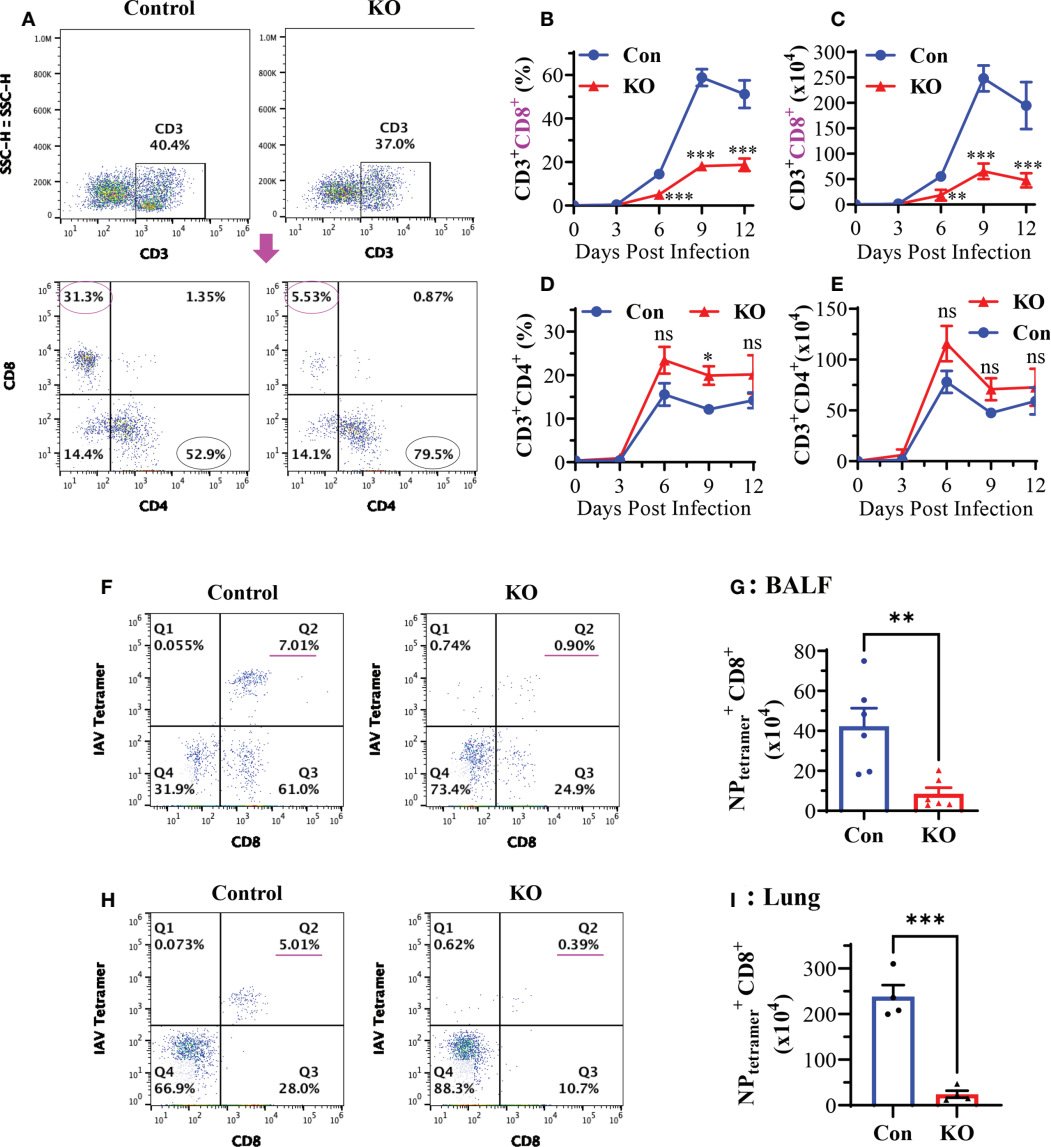
KO of *Runx3* in adult mice profoundly reduces CD8^+^ cytotoxic T cells in IAV-infected lungs. Sex- and age-matched littermate control (Con) and *Runx3* KO (KO) mice were infected with 1 LD_50_ of H1N1 PR8 strain, and BALFs were collected and subjected to flow cytometry analysis. **(A–E)** KO of *Runx3* markedly reduces total pulmonary CD8^+^ cytotoxic T cells in response to IAV infection. CD8^+^ and CD4^+^ T cells were identified based on the selected surface markers from the CD3^+^-gated cell populations, and the cell frequencies **(B, D)** and absolute numbers **(C, E)** were shown in the bar graphs. A representative FACS plot evaluated on day 6 pi is shown in **(A)**. Results are presented as means ± SE. Days 0 and 3 pi, n = 3; days 6 to 12 pi, n = 6-10. **(F–I)** Profound reduction of pulmonary NP-specific CD8^+^ cytotoxic T cells by *Runx3* KO. H2D^b^ MHC class I tetramer of IAV NP_366-374_ and CD8 antibody were used to identify the NP_366-374_-specific CD8^+^ effector T cells( ${\text{NP}}_{\text{tetramer}}^{+}{CD8}^{+}$ ) in BALFs (F-Q2) and whole lungs (H-Q2) as shown in representative FACS plots evaluated on day 9 pi. The ${\text{NP}}_{\text{tetramer}}^{+}{CD8}^{+}$ T cell numbers in BALFs **(G)** and whole lungs **(I)** were shown in bar graphs. Results are presented as means ± SE (n = 4-6). Two-way ANOVA and unpaired Student’s t test were performed in **(B–E)** and unpaired Student’s t test was performed in **(G, I)**. *p<0.05; **p<0.01; ***p<0.001 vs control mice. NS, not statistically significant.

Pulmonary CD8^+^ cytotoxic T cells play a central role in the clearance of virus after primary IAV infection (7, 36, 37). We assessed the effect of *Runx3* KO on the generation of pulmonary CD8^+^ cytotoxic T cells in IAV-infected mouse lungs. Flow cytometry analyses of BALF cells revealed that CD8^+^ effector T cell frequency and absolute numbers were profoundly reduced by *Runx3* KO during IAV infection (**Figures 5A–C**). Whereas, pulmonary CD4^+^ T cell frequency and absolute numbers tended to increase but without statistically significant (**Figures 5D, E**). We further found that the frequency and populations of IAV NP_366-374_-specific CD8^+^ cytotoxic T cells in BALFs and whole lungs were reduced more than 85% by *Runx3* KO on day 9 post IAV infection (**Figures 5F–I**). Thus, our findings indicate that Runx3 is essential for the generation of lung CD8^+^ cytotoxic T cells against IAV infection.

### General KO of *Runx3* augments lung accumulation of innate immune cells and pro-inflammatory cytokines by IAV infection

**Figure 6:**
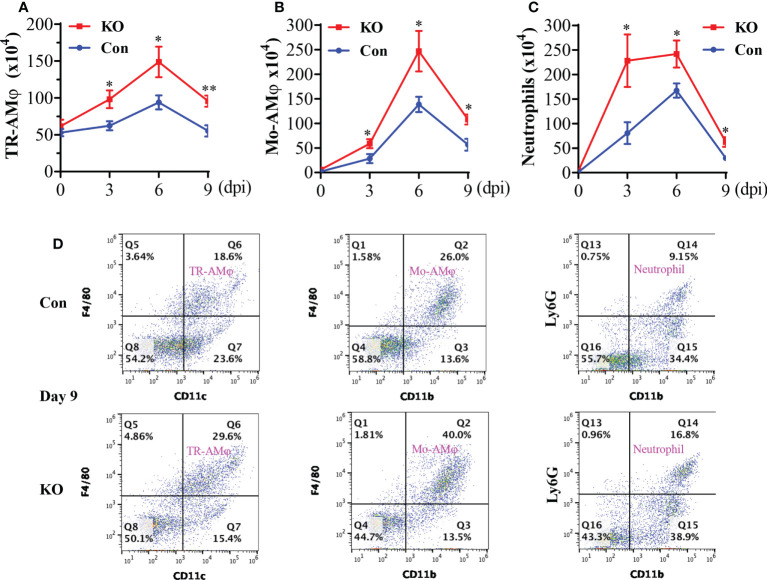
A general *Runx3* KO in adult mice augments lung accumulation of innate immune cells in response to IAV Infection. Sex-and age-matched littermate control (Con) and *Runx3* KO mice were infected with 1 LD_50_ of H1N1 PR8 strain and BALFs were collected and subjected to flow cytometry analysis. TR-AMφ **(A)**, Mo-AMφ **(B)**, and neutrophils **(C)** were identified based on the selected surface markers and the cell absolute numbers were shown in bar graphs **(A–C)**. Results are presented as means ± SE. TR-AMφ, n = 5-7; Mo-AMφ, n = 5-6; neutrophils, n = 5-6. Unpaired Student’s t test was performed in **(A–C)** *p<0.05; **p<0.01 vs control mice. **(D)** Representative FACS plots of BALF innate immune cells evaluated day 9 post IAV infection. KO of *Runx3* markedly augmented pulmonary accumulation of TR-AMφ, Mo-AMφ, and neutrophils in response to IAV infection.

**Figure 7:**
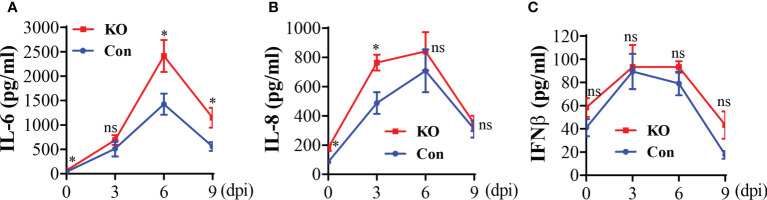
A general *Runx3* KO in adult mice increases lung production of pro-inflammatory cytokines in response to IAV Infection. Sex-and age-matched littermate control (Con) and *Runx3* KO mice were infected with 1 LD_50_ of H1N1 PR8 strain and BALFs were collected and subjected to ELISA for IL-6 **(A)**, IL-8 **(B)** and IFNβ **(C)**. Results are presented as means ± SE. IL-6, n = 5-8; IL-8; n = 6-9; IFNβ, n = 7. Unpaired Student’s t test was performed in **(A–C)** *p<0.05 vs control mice. NS, no significance.

The aforementioned data suggest that a general *Runx3* KO may activate other host antiviral immune pathways to compensate for the loss of pulmonary CD8^+^ cytotoxic T cells. We collected BALF immune cells and determined innate immune cell types by flow cytometer analysis. We found that *Runx3* KO significantly increased the absolute numbers of tissue resident alveolar macrophages (TR-AMφ, F4/80^+^CD11c^+^) (38, 39) during IAV infection (**Figures 6A, D**-left panels). General *Runx3* KO also markedly augmented pulmonary recruitment and accumulation of monocyte-derived alveolar macrophages (Mo-AMφ, F4/80^+^CD11b^+^) (38, 39) and neutrophils (Ly6G^+^CD11b^+^) during IAV infection (**Figures 6B–D**). Moreover, we found that *Runx3* KO significantly enhanced BALF levels of IL-6 and IL-8 on days 6 and 9 or day 3 post IAV infection, respectively (**Figures 7A, B**). KO of *Runx3* also significantly increased BALF levels of IL-6 and IL-8 under normal conditions (day 0). Whereas, KO of *Runx3* appeared to enhance lung production of IFNβ during IAV infection compared with littermate control mice, but the effect was not statistically significant (**Figure 7C**). These data suggest that a general KO of *Runx3* may activate aspects of host innate immunity against IAV infection.

### Runx3 is strongly detected in CCR2^+^ immune cells in IAV-infected mouse lungs and is induced in activated macrophages and DCs

**Figure 8:**
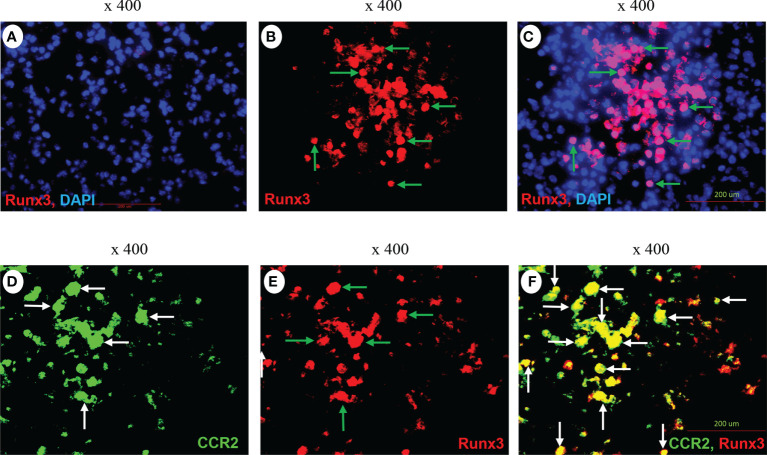
Immunofluorescence detection of Runx3 co-localization with CCR2^+^ immune cells in IAV-infected mouse lungs. **(A–C)** Immunofluorescence (IF) detection of Runx3 in IAV-infected mouse lungs. Lung sections from saline-treated (Mock, **A**) or H1N1-infected mice on day 7 pi **(B,C)** were subjected to IF staining with a Runx3 monoclonal antibody followed by Alexa Fluor 568-labelled goat anti-mouse secondary IgG (n = 6/group). Runx3 immunoreactivities are stained red (**B**, green arrows), cell nuclei stained blue by DAPI, and merged pink **(C)**. **(D–F)** Co-localization of Runx3 with CCR2^+^ immune cells in IAV-infected mouse lungs. Lung sections from H1N1-infected mice were subjected to IF staining with a FITC-labeled CCR2 polyclonal antibody and a Runx3 monoclonal antibody followed by Alexa Fluor 568-labelled goat anti-mouse secondary IgG (n = 4). CCR2 is stained green (**D,** white arrows), Runx3 red (**E,** green arrows), and merged yellow (**F,** white arrows).

**Figure 9:**
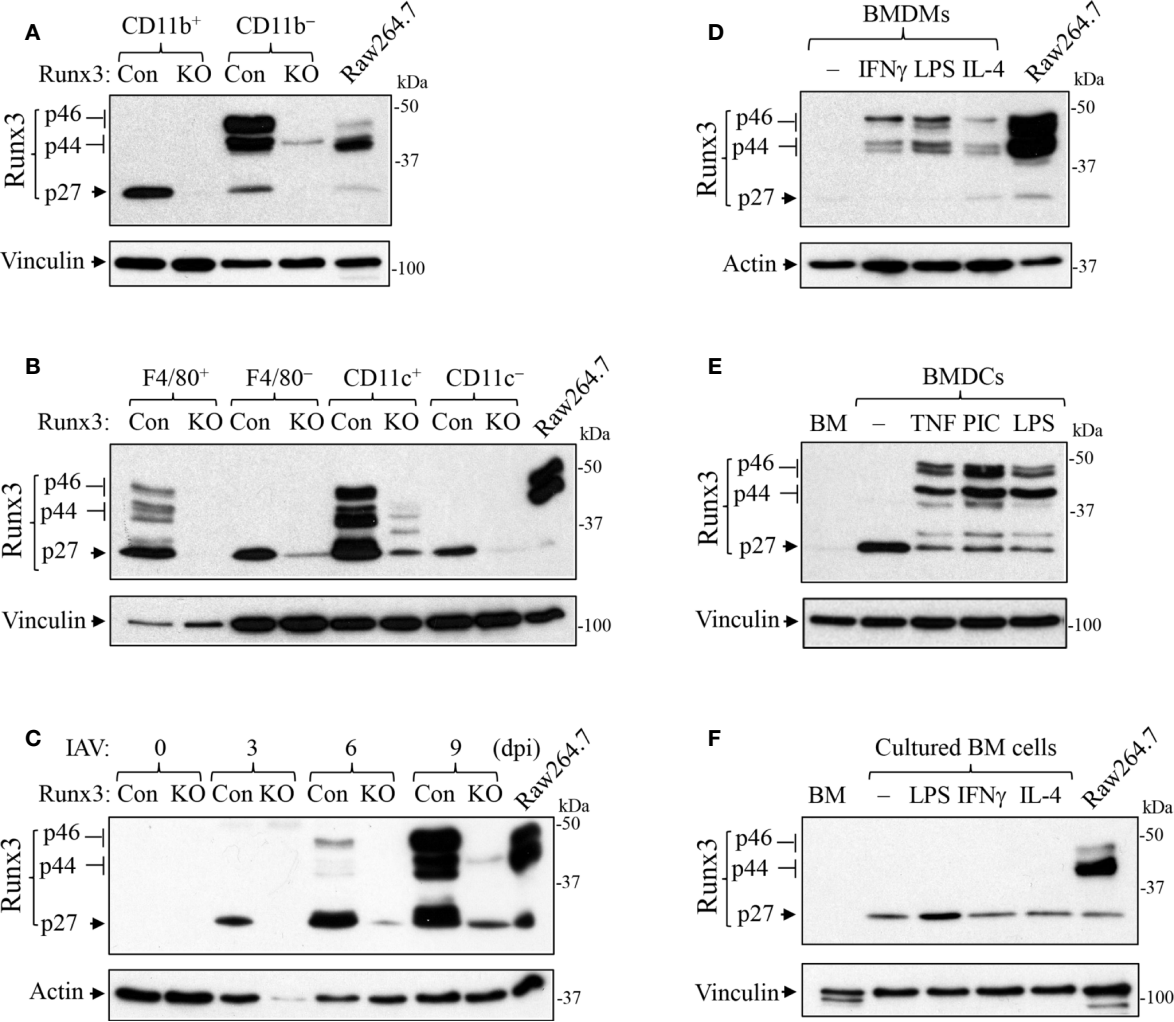
Runx3 is induced in activated macrophages and DCs. **(A, B)** Runx3 expression in the isolated lung innate immune cells from IAV-infected mouse lungs on day 5 pi. Equal amounts of cell lysates except that of F4/80^+^ cells were subjected to Western blotting with Runx3 antibody. **(C)** Runx3 is gradually induced in BALF cells from control mice during IAV infection. Control (Con) and *Runx3* KO mice were infected with 1 LD_50_ of H1N1 PR8 strain, and BALF cells were collected at different days post infection (dpi). Equal amounts of cell lysates were subjected to Western blotting with Runx3 antibody. **(D–F)** Runx3 is induced in activated macrophages and DCs. BMDMs **(D)**, BMDCs **(E)**, and cultured bone marrow (BM) cells **(F)** were treated without (−) or with IFNγ (25 ng/ml), LPS (100 ng/ml), IL-4 (10 ng/ml), TNFα (10 ng/ml), or poly(I:C) (PIC, 1 μg/ml) for 24 h. Equal amounts of cell lysates were subjected to Western blotting with indicated antibodies. BM, freshly isolated bone marrow. Raw264.7, mouse macrophage cell line. Results are representative of the findings of three independent experiments.

We performed immunofluorescence analysis and found that Runx3 was barely detected in mock control mouse lungs (**Figure 8A**) but was strongly expressed in IAV-infected mouse lungs (**Figure 8B**). Runx3 immunoreactivity (red) essentially merged with DAPI (blue) to form pink color signals (**Figures 8B, C**), indicating the cellular nuclear localization of Runx3. Double immunofluorescence analysis showed that the immunoreactivity of a hematopoietic immune cell biomarker CCR2 (green) strongly merged with Runx3 staining (red) to form yellow color signals (**Figures 8D–F**). These results indicate that Runx3 is strongly detected in CCR2^+^ immune cells in IAV-infected mouse lungs. As CCR2 is highly expressed in monocytes and monocyte-derived macrophages and DCs (40), we determined the expression of Runx3 in the cells. Three Runx3 isoforms (p46, p44 and p27) have been detected and the p44/p46 isoforms are normally seen in matured leukocytes (14). The p27 isoform lacks half of the DNA binding domain and part of the transactivation region. Among the isolated lung innate immune cells from IAV-infected mouse lungs on day 5 pi, we found that only the p27 immature Runx3 isoform was expressed in lung CD11b^+^ infiltrating immune cells such as monocytes and neutrophils. Whereas, the p44/p46 Runx3 mature isoforms were predominantly expressed in CD11b^−^ cells from control mouse lungs (**Figure 9A**). We further found that all three Runx3 isoforms were expressed in pulmonary F4/80^+^ macrophages and CD11c^+^-expressing DCs and resident alveolar macrophages isolated from IAV-infected control mice (**Figure 9B**). Pulmonary F4/80^−^ and CD11c^−^ immune cells mainly expressed the p27 Runx3 isoform on day 5 pi. Moreover, we found that all three Runx3 isoforms were gradually induced in BALF cells from control mice during IAV infection, reaching a peak on day 9 pi (**Figure 9C**). Noticeably, all the Runx3 protein bands were essentially depleted in the pulmonary immune cells isolated from IAV-infected *Runx3* KO mice (**Figures 9A–C**), confirming the successful deletion of *Runx3* in the inducible KO mouse model. We next determined if Runx3 could be induced in macrophages and DCs activated by M1 (IFNγ, LPS, TNFα, or dsRNA poly(I:C)) or M2 (IL-4) macrophage stimulators (35). As shown in **Figures 9D–F**, the p27 immature Runx3 isoform was barely detected in unstimulated BMDMs and freshly isolated BM cells but was expressed in cultured BM cells and abundant in unstimulated BMDCs. Interestingly, the p44/p46 Runx3 mature isoforms were strongly induced by IFNγ, LPS, IL-4, TNFα, and dsRNA poly(I:C) in both BMDMs and BMDCs (**Figures 9D, E**). In contrast, the p44/p46 Runx3 isoforms could not be induced by the stimuli in BM cells (**Figure 9F**). These findings indicate that Runx3, especially the p44/p46 isoforms, are markedly induced in the differentiated and activated macrophages and DCs, but not in BM progenitor cells. The upregulation of Runx3 in macrophages and DCs could play a role in host immune response to IAV infection.

## Discussion

In this study, we have generated an inducible *Runx3* global KO mouse model and shown that *Runx3* KO in adult C57BL/6 mice does not affect animal survival at least 250 days post deletion. Compared to control mice, we only observed that mice gained less weight on day 9 for male mice and from days 9 to 20 for female mice post *Runx3* ablation. Previous studies show that Runx3 is required for embryonic development and together with Runx1 is involved in thymopoiesis (13). A general *Runx3* KO mice on the background of C57BL/6 and BALB/c died soon after birth (15, 16). However, T or NK cell type-specific *Runx3* KO mice were viable (20–22). We further found that inducible *Runx3* KO in adult mice did not significantly affect thymus weight, the frequency and absolute populations of single CD4^+^ or CD8^+^ positive T cells in thymus. Hence, our findings indicate that a general *Runx3* KO in adult mice has a minor effect on animal survival and thymic T cell generation under normal conditions.

In mouse, the expression of Runx3 is normally confined to leukocytes but not epithelium (11, 41). By using T cell-specific *Runx3* KO adult mice, it has been demonstrated that Runx3 is required for the recruitment, differentiation and local expansion of CD8^+^ T effector/memory cells and for the virus clearance in response to LCMV infection (21, 23). Runx3 is also required for the differentiation and local expansion of NK cells in response to mouse cytomegalovirus infection, which was demonstrated by using NK cell-specific *Runx3* KO mouse model (22). Here, we provided compelling evidence that a general KO of *Runx3* in adult mice resulted in a huge reduction (>85%) in numbers of total and IAV NP-specific pulmonary CD8^+^ cytotoxic T cells during IAV infection, while it had a minor effect on pulmonary generation of CD4^+^ T cells. To our surprise, we found that a general KO of *Runx3* did not significantly altered IAV viral clearance, which is in contrast to the outcome of LCMV infection observed in T cell-specific *Runx3* KO mice (21). Instead, a general *Runx3* KO slightly reduced mortality by IAV infection compared with littermate control mice, although the effect was not statistically significant. As pulmonary CD8^+^ cytotoxic T cells play a central role in the clearance of virus after primary IAV infection (7, 36, 37), we infer that general *Runx3* KO may activate other host antiviral immune pathways to compensate for the loss of pulmonary CD8^+^ cytotoxic T cells during IAV infection. Indeed, we found that *Runx3* general KO significantly increased the numbers of pulmonary innate immune cells such as macrophages and neutrophils and the production of pro-inflammatory cytokines during IAV infection.

In contrast to CD8^+^ T and NK cells, several lines of evidence indicate that Runx3 functions as a repressor in DCs. It has been shown that *Runx3* KO promoted DC maturation and its potency to stimulate CD4^+^ T cell proliferation *in vitro* (18). KO of *Runx3* also promoted pulmonary DC migration to thoracic lymph nodes by LPS treatment (19). Moreover, KO of *Runx3* in plasmacytoid DCs (pDCs) promoted DC maturation by CpG treatment and enhanced skin fibrosis by bleomycin in mice (24). We found that Runx3 was strongly detected in CCR2^+^ immune cells in IAV-infected mouse lungs and was induced in activated macrophages and DCs. Specifically, the p27 immature Runx3 isoform was expressed in unstimulated BMDCs and lung CD11b^+^ infiltrating immune cells, such as monocytes and neutrophils. The p44/p46 Runx3 mature isoforms were strongly induced in inflammatory mediator-activated BMDMs and BMDCs and highly expressed in pulmonary F4/80^+^ macrophages and CD11c^+^-expressing DCs and resident alveolar macrophages isolated from IAV-infected control mice. Moreover, we found that all three Runx3 isoforms were gradually induced in BALF cells from control mice during IAV infection, reaching a peak on day 9 pi. These findings suggest that the induction of Runx3 in activated macrophages and DCs may restrain host innate and/or adaptive immune responses to IAV infection. Deficiency of *Runx3* in the cells may augment host innate and/or adaptive immunities for combating IAV infection, hence compensating for the loss of pulmonary CD8^+^ cytotoxic T cells during IAV infection. This hypothesis merits further investigation by using macrophage- and DC-specific *Runx3* KO mouse models, which are under development in our laboratory. A more recent study indicates that KO of *Runx3* in mouse colonic CD11c-expressing cells, including resident macrophages and DCs, suppressed the expression of TGFβ-regulated genes and ß-catenin signaling associated genes respectively, leading to up-regulation of pro-inflammatory genes (42). This effect likely is the potential mechanism for the pro-inflammatory activation of lung innate immune cells in *Runx3* KO mice in response to IAV infection. In addition, it is possible that other mechanisms such as the modulation of type-I IFN signaling as proposed by Runx1 in A549 cells (28) may likewise contribute to the enhanced anti-IAV activities in *Runx3* general KO mice.

In summary, we have generated an inducible *Runx3* general KO mouse model and shown that *Runx3* KO in adult mice has a minor effect on animal survival and thymic function in normalcy. We further show that a general KO of *Runx3* in adult mice results in a huge reduction (>85%) in numbers of total and antigen-specific pulmonary CD8^+^ cytotoxic T cells, while it significantly increases the numbers of pulmonary innate immune cells such as macrophages and neutrophils during IAV infection. As a result, a general KO of *Runx3* did not significantly affect animal outcomes including viral clearance and animal survival in response to IAV infection. Our findings suggest that *Runx3* KO may enhance host innate immunity to compensate for the loss of pulmonary CD8^+^ cytotoxic T cells during IAV infection.

## Data availability statement

The original contributions presented in the study are included in the article/supplementary material. Further inquiries can be directed to the corresponding author.

## Ethics statement

The animal study was reviewed and approved by Institutional Animal Care and Use Committee at the University of Texas Health Science Center at Tyler.

## Author contributions

QH, SK, and HT conceived and designed the study. QH, SK, and HT performed the experiments and analyzed the data. HT wrote and edited the manuscript. SS, TT, and SI provided reagents and data interpretation and edited the manuscript. All authors read and approved the manuscript.

## Funding

This study was supported by a NIH grant AI128442 (to HT).

## Conflict of interest

The authors declare that the research was conducted in the absence of any commercial or financial relationships that could be construed as a potential conflict of interest.

## Publisher’s note

All claims expressed in this article are solely those of the authors and do not necessarily represent those of their affiliated organizations, or those of the publisher, the editors and the reviewers. Any product that may be evaluated in this article, or claim that may be made by its manufacturer, is not guaranteed or endorsed by the publisher.
